# NLRC4 Inflammasome-Driven Immunogenicity of a Recombinant MVA Mucosal Vaccine Encoding Flagellin

**DOI:** 10.3389/fimmu.2017.01988

**Published:** 2018-01-24

**Authors:** Stephanie L. Sanos, Ronny Kassub, Marco Testori, Marlene Geiger, Juliane Pätzold, Raphael Giessel, Johanna Knallinger, Barbara Bathke, Fabienne Gräbnitz, Kay Brinkmann, Paul Chaplin, Mark Suter, Hubertus Hochrein, Henning Lauterbach

**Affiliations:** ^1^Bavarian Nordic GmbH, Martinsried, Germany; ^2^University of Zurich, Zurich, Switzerland

**Keywords:** mucosal vaccines, MVA, flagellin, inflammasome, intestinal mucosa

## Abstract

Bacterial flagellin enhances innate and adaptive immune responses and is considered a promising adjuvant for the development of vaccines against infectious diseases and cancer. Antigen-presenting cells recognize flagellin with the extracellular TLR5 and the intracellular NLRC4 inflammasome-mediated pathway. The detailed cooperation of these innate pathways in the induction of the adaptive immune response following intranasal (*i.n*.) administration of a recombinant modified vaccinia virus Ankara (rMVA) vaccine encoding flagellin (rMVA-flagellin) is not known. rMVA-flagellin induced enhanced secretion of mucosal IL-1β and TNF-α resulting in elevated CTL and IgG2c antibody responses. Importantly, mucosal IgA responses were also significantly enhanced in both bronchoalveolar (BAL) and intestinal lavages accompanied by the increased migration of CD8^+^ T cells to the mesenteric lymph nodes (MLN). *Nlrc4^−/−^* rMVA-flagellin-immunized mice failed to enhance pulmonary CTL responses, IgG2c was lower, and IgA levels in the BAL or intestinal lavages were similar as those of control mice. Our results show the favorable adjuvant effect of rMVA-flagellin in the lung as well as the intestinal mucosa following *i.n*. administration with NLRC4 as the essential driver of this promising mucosal vaccine concept.

## Introduction

After breaching mucosal surfaces, the innate immune system is the first line of defense against invading pathogens. It is activated following the engagement of germ-line encoded pattern-recognition receptors (PRRs) expressed on innate immune cells with unique microbial components, pathogen-associated-molecular patterns or endogenous damage-associated molecular patterns. Families of PRRs include among others membrane-bound toll-like receptors (TLRs) (TLR1, 2, 4, 5, 6, and 10), those found in the endosomal compartment (TLR3, 7, 8, 9, 11, 12, and 13) (1), RNA-sensing RIG-like helicases (RIG-I, MDA-5) (2), DNA sensors AIM2, LRRFIP1, DAI (3, 4), and Nod-like-receptors (NLR), the main component in the inflammasome complex (5, 6).

Inflammasomes are protein platforms regulating caspase-1 activation for the production of IL-1β and IL-18 (5). Caspase-1 also induces an inflammatory form of cell death, known as pyroptosis. The NLRP3 inflammasome responds to pathogen-derived molecules from bacteria, viruses, fungi, in addition to host-derived molecules of stress and environmental irritants (6). Bacterial flagellin was initially described to bind and activate TLR5, resulting in a MyD88-dependent release of proinflammatory cytokines (7). Later, it was recognized that cytosolic flagellin can also be sensed by the NAIP5/NLRC4 inflammasome (5) and NAIP5’s function is to link flagellin to NLRC4 (8).

Studies have described flagellin as a potent adjuvant, in the context of a broad range of recombinant vaccines (9). Its effect occurs on various antigen-presenting cells, airway structural epithelial cells (10) and enterocytes (11). Flagellin is currently examined in a wide range of vaccines, including mucosal vaccines being used separately with the antigen or commonly administered as fusion proteins (12–16). Flagellin is also used as an adjuvant in the development of a therapeutic vaccine against certain types of cancer and was described as a strong vaginal adjuvant in a model of genital cancer (17) and a murine melanoma model (18). As well as inducing innate immune responses, flagellin exerts effects on adaptive immunity by inducing the proliferation of antigen-specific CD4^+^ T cells (9), together with robust antibody responses.

Viral vectors based on poxvirus family members have proven to be efficient and safe inducers of strong and sustained B and T cell responses. The licensed third-generation smallpox vaccine, MVA-BN^®^, is a highly attenuated orthopoxvirus exhibiting an excellent safety profile (19). Simultaneously, MVA-based viral vectors expressing heterologous antigens induce strong humoral and cellular immune responses against foreign antigens (20). Therefore, MVA is a potent and safe viral vector for the development of vaccines against infectious diseases and for cancer immunotherapy. Moreover, the mucosal application of MVA has previously been described where *i.n*. delivery of MVA led to the development T and B cell responses (21, 22).

In an attempt to combine the immunogenicity of viral vectors, the safety of MVA and the unique property of flagellin, we have generated a recombinant (r)MVA-encoding flagellin from *Salmonella typhimurium* and the model antigen OVA. We report that *i.n*. immunization with rMVA-flagellin elicits enhanced cellular and humoral immune responses, both systemically and at mucosal sites. More interestingly, we could also show for the first time that *i.n*. immunization with rMVA-flagellin could elicit gastro-intestinal (GI) immune responses. We also describe the role of NLRC4 in driving rMVA-flagellin specific mucosal humoral responses. This novel approach makes rMVA-flagellin a candidate for the development of a mucosal vaccine against a broader range of diseases including GI and respiratory pathogens.

## Results

### Innate Immune Response in the Bronchoalveolar (BAL) of *i.n*. rMVA-Flagellin-Immunized Mice

We designed a genetically modified rMVA encoding the model antigen OVA and flagellin (rMVA-OVA-flagellin), with the aim to further improve immunogenicity upon *i.n*. application of our rMVA vaccine, also encoding OVA (rMVA-OVA). Since the priming of adaptive immune responses depends on innate recognition of the vaccine, we addressed the innate response induced after *i.n*. administration of our novel vaccine.

**Figure 1:**
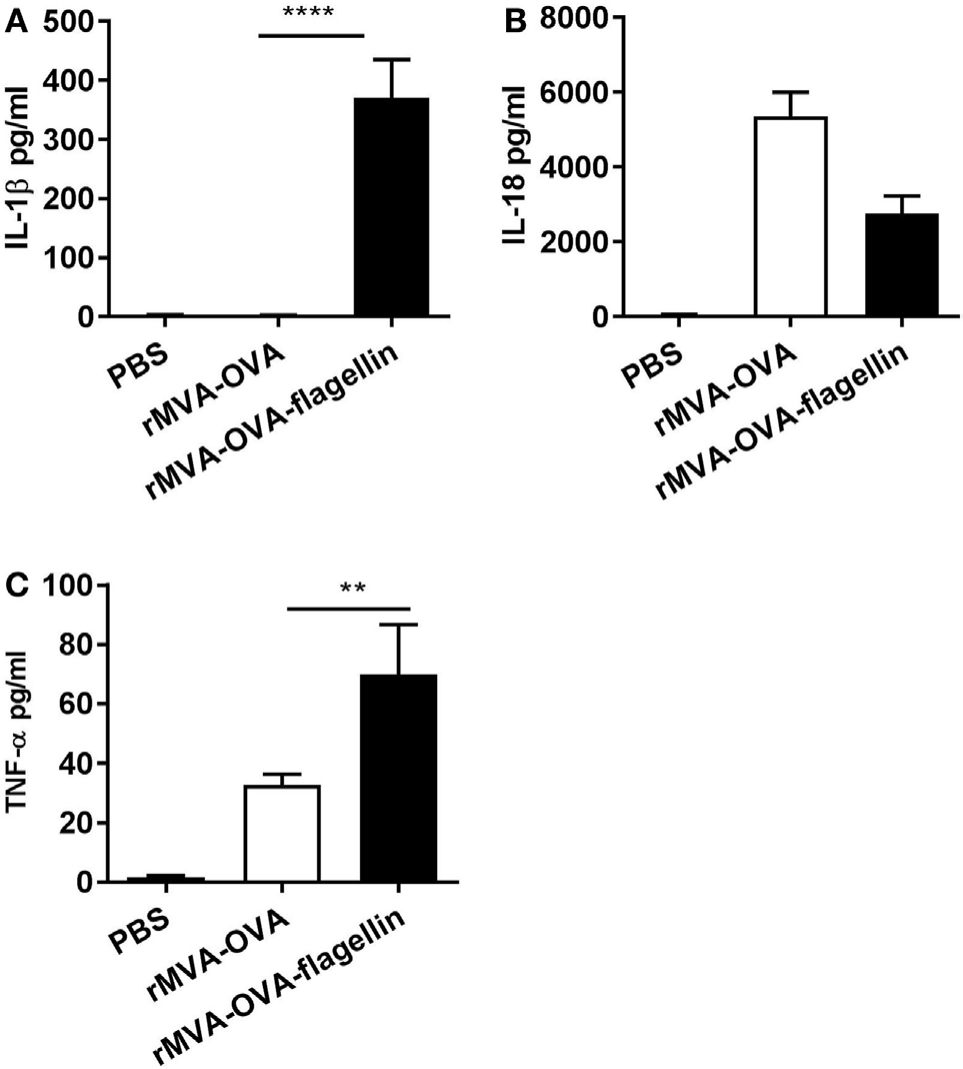
rMVA vaccine, also encoding OVA (rMVA-OVA)-flagellin enhances lung innate immune responses. Mice were immunized *i.n*. with rMVA-OVA, rMVA-OVA-flagellin, or PBS. 24 h following immunization, bronchoalveolar (BAL) was collected, and IL-1β **(A)**, IL-18 **(B)**, TNF-α **(C)**, cytokine production was determined by Luminex. Results represent the mean of individual BAL (16–20 mice) combined from six independent experiments and are shown as mean (±SEM). Statistical analyses were done by one-way ANOVA **p < 0.005, ****p < 0.0001.

Following *i.n*. immunization with rMVA-OVA-flagellin, cytokine production was measured in the BAL. Previous kinetic experiment (3, 6, 12, and 24 h) demonstrated an optimal cytokine response at a 24-h time-point (data not shown), which was, therefore, chosen for the study. Our results show that inflammasome-specific cytokines IL-1β (Figure 1A) and IL-18 (Figure 1B) could be detected. Mice immunized with rMVA-OVA also exhibited production of IL-18 (Figure 1B), but no IL-1β (Figure 1A).

Enhanced TNF-α could be observed in rMVA-OVA-flagellin-immunized mice compared to rMVA-OVA (Figure 1C), suggesting a TLR-mediated innate immune sensing. The levels of IL-6 were similar after rMVA-OVA and rMVA-OVA-flagellin immunization (data not shown).

Together, these results indicate that *i.n*. rMVA-OVA-flagellin immunization *in vivo* can trigger both inflammasome and TLR-mediated innate immune responses.

### IL-1β Is Partially Produced by Mucosal DCs

Myeloid cells, including DCs, macrophages, or neutrophils, are an important source of IL-1β. Having established IL-1β production in the BAL of rMVA-OVA-flagellin-immunized mice, we addressed the potential *in vivo* contribution of DCs in our vaccination model.

**Figure 2:**
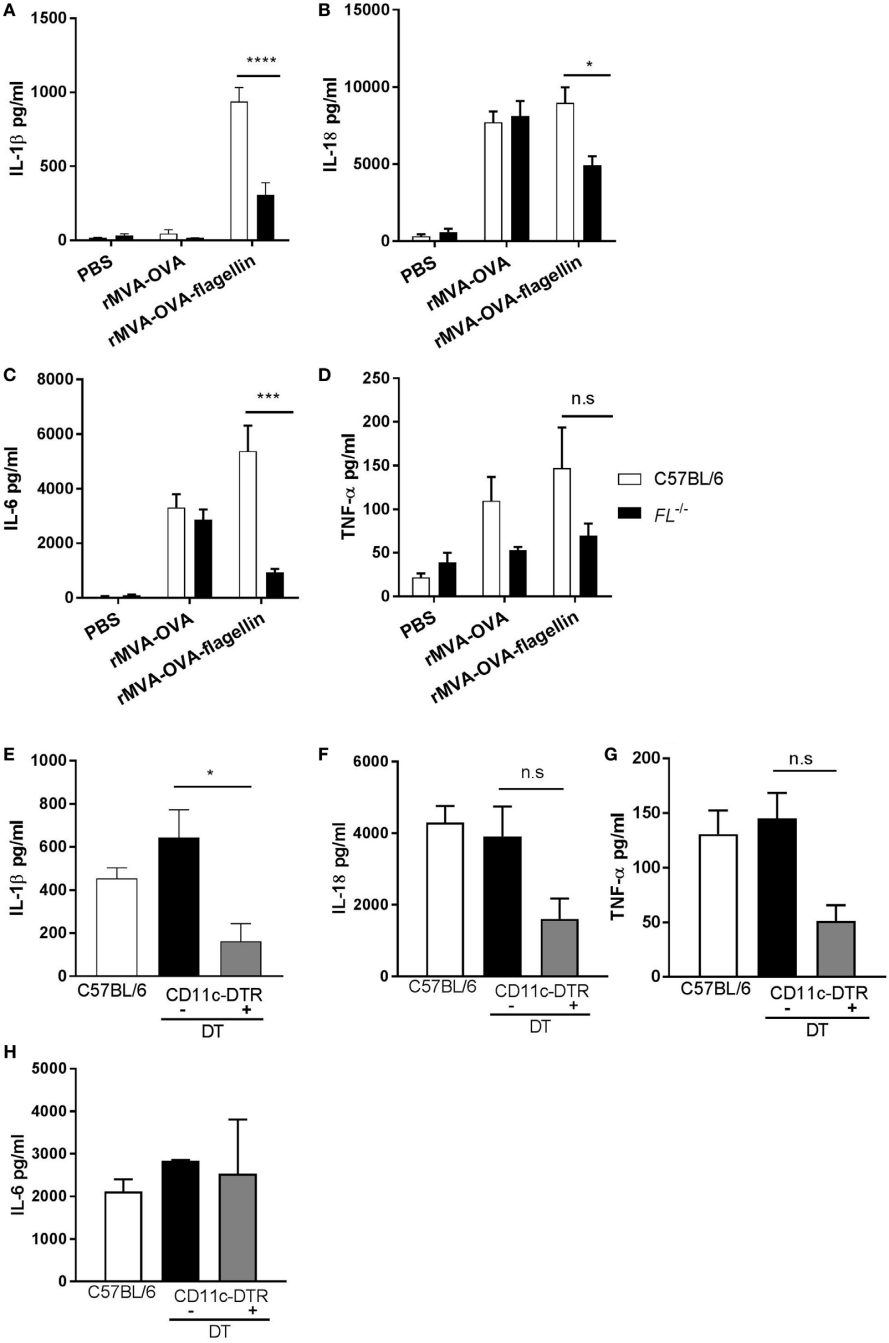
IL-1β is mainly produced by lung DCs after *i.n*. rMVA vaccine, also encoding OVA (rMVA-OVA)-flagellin immunization. All mice were immunized *i.n*. with rMVA-OVA, rMVA-OVA-flagellin, PBS **(A–D)**, or rMVA-OVA-flagellin only **(E–H)**. **(A–D)** C57BL/6, *FL^−^*^/^*^−^*, **(E–H)** CD11c-DTR, and CD11c-DTR mice treated with DT a day prior to immunization. 24 h following immunization, bronchoalveolar (BAL) was collected and IL-1β, IL-6, IL-18, and TNF-α production was measured with Luminex. Results represent the mean of individual BAL (3–9 mice) and are shown as mean (±SEM). Statistical analyses were done by one-way ANOVA **(E–H)** or two-way ANOVA **(A–D)**: *p < 0.05, ***p < 0.0005, ****p < 0.0001.

Two different models were used, where the *in vivo* role of DCs could be investigated. First, rMVA-OVA-flagellin-immunized *Flt3-L^−/−^* mice, known to harbor drastically reduced numbers of dendritic cells, exhibited a decrease of IL-1β compared to wt mice (wt: 940 ± 85 pg/ml; *Flt3-L^−/−^*: 305 ± 75 pg/ml) (Figure 2A). Similar decrease of IL-18 (Figure 2B) and IL-6 (Figure 2C) could be observed, although never abrogated, whereas TNF-α (Figure 2D) was unaffected. Next, we investigated conditional ablation of DCs using *CD11c*-DTR mice. In order to deplete lung DCs, *CD11c*-DTR mice were treated with DT *i.n*. prior to immunization with rMVA-OVA-flagellin. In our model, we could observe that DT treatment led to the depletion of the CD103^+^CD11b^−^ DC subset (Figure S1 in Supplementary Material). BAL of DT-treated *CD11c*-DTR mice exhibited significant reduction of IL-1β compared to non-depleted controls (*CD11c*-DTR + PBS: 644 ± 130 pg/ml; *CD11c*-DTR + DT 256 ± 116 pg/ml) (Figure 2E), whereas IL-18 (Figure 2F), TNF-α (Figure 2G), and IL-6 (Figure 2H) were unchanged after DT treatment.

Altogether, these data indicate that mucosal DCs are a major source of IL-1β, in response to *i.n*. MVA-flagellin immunization.

### *In Vitro* Inflammasome and TLR-Mediated Recognition by DC

Having described the *in vivo* role of DCs in rMVA-OVA-flagellin lung innate immune recognition, we investigated cytokine production of *in vitro* bone marrow (BM) derived DCs cultured in the presence of Flt3-L (FL) (FL-DCs) transduced with rMVA-OVA, rMVA-OVA-flagellin or recombinant flagellin.

**Figure 3:**
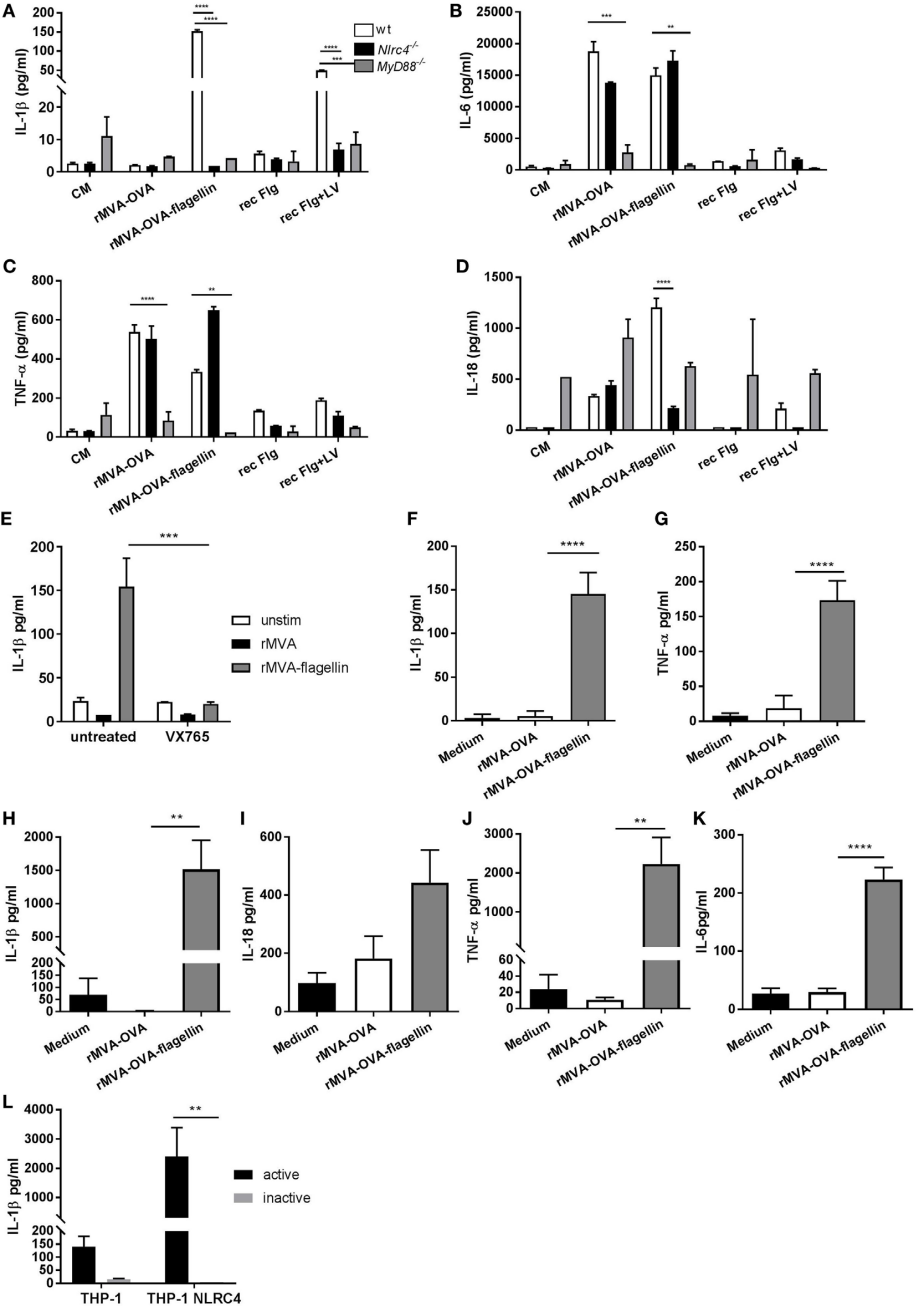
*In vitro* stimulation of FL-DCs, THP-1, and THP-1-overexpressing NLRC4 with rMVA vaccine, also encoding OVA (rMVA-OVA)-flagellin induces NLRC4 and toll-like receptor (TLR)-specific cytokines. BM-derived wt, *Myd88^−/−^* and *Nlrc4^−/−^* FL-DCs **(A–D)** were stimulated with rMVA-OVA, rMVA-OVA-flagellin, flagellin alone or flagellin and Lyovec. **(E)** FL-DCs were pre-treated with the caspase-1 inhibitor VX-675, and stimulated with rMVA-OVA or rMVA-OVA-flagellin. **(F,G)** THP-1 and **(I–K)** THP-1-NLRC4 cells were stimulated with rMVA-OVA and rMVA-OVA-flagellin. **(L)** THP-1 and THP-1-NLRC4 cells were stimulated with active and inactive rMVA-OVA-flagellin virus. Cells were cultured overnight for 24 h, and the supernatant was collected. IL-1β **(A,E,F,H)**, IL-18 **(D,I)**, IL-6 **(B,K)**, and TNF-α **(C,G,J)** production was measured with Luminex. Data are shown as mean (±SEM) and is representative of three independent experiments. Statistical analyses were done by one-way ANOVA **(F–K)** or two-way ANOVA **(A–E,L)**: **p < 0.005, ***p < 0.0009, ****p < 0.0001.

IL-1β was solely produced from rMVA-OVA-flagellin-stimulated FL-DCs, with marginal secretion following cytosolic delivery of recombinant flagellin (Figure 3A). This response was completely abrogated in *Nlrc4^−/−^* FL-DCs. Similarly, the IL-18 response in rMVA-OVA-flagellin-stimulated FL-DCs was also strongly reduced in *Nlrc4^−/−^* FL-DCs (Figure 3D), but unaffected in rMVA-OVA-stimulated wt and *Nlrc4^−/−^* FL-DCs. This highlights the role of the NLRC4 inflammasome in MVA-OVA-flagellin-induced IL-1β and IL-18 production from DCs. When we investigated the cytokines IL-6 (Figure 3B) and TNF-α (Figure 3C), both cytokines were produced from rMVA and rMVA-flagellin-stimulated FL-DCs. *MyD88^−/−^* FL-DCs, unable to respond to various TLRs including TLR5, stimulated with rMVA-OVA-flagellin showed significant reduction of IL-6 (Figure 3B) and TNF-α (Figure 3C), while still produced in *Nlrc4^−/−^* FL-DCs, confirming the role of the TLR pathway in rMVA-OVA-flagellin innate immune recognition by DCs. Moreover, secretion of active IL-1β after NLRC4 activation requires caspase-1. Therefore, we investigated the role of caspase-1 in rMVA-OVA-flagellin-stimulated wt FL-DCs treated with the caspase-1 inhibitor, VX-765. A clear inhibition of IL-1β was observed (Figure 3E), suggesting inflammasome-dependent caspase-1 activation in response to rMVA-OVA-flagellin.

To elucidate if the differential detection of flagellin in MVA *via* TLR and inflammasome-dependent ways, described above for murine cells, could also be found for human cells, the human monocytic cell line, THP-1, known to naturally express moderate levels of TLR5 (23) was used. Notably, rMVA-OVA-flagellin induced highest levels of IL-1β (Figure 3F) and TNF-α (Figure 3G) in THP-1 cells. To confirm the role of NLRC4, we employed THP-1 cells with NLRC4 overexpression. Indeed, these cells produced elevated levels of IL-1β (Figure 3H), IL-18 (Figure 3I), TNF-α (Figure 3J), and IL-6 (Figure 3K) when they were stimulated with rMVA-OVA-flagellin but not rMVA-OVA. This response was fully dependent on the genetic activity of the virus since the induction of IL-1β was completely lost upon heat-inactivation of the MVA-OVA-flagellin (Figure 3L).

These data indicate both a role of the inflammasome and TLR pathway in innate immune recognition of rMVA-OVA-flagellin by DCs, together with cytosolic delivery of flagellin essential for optimal cytokine responses. Importantly, the data are consistent in both murine and human cells.

### NLRC4-Mediated IL-1β Lung Innate Immune Responses in rMVA-Flagellin-Immunized Mice

**Figure 4:**
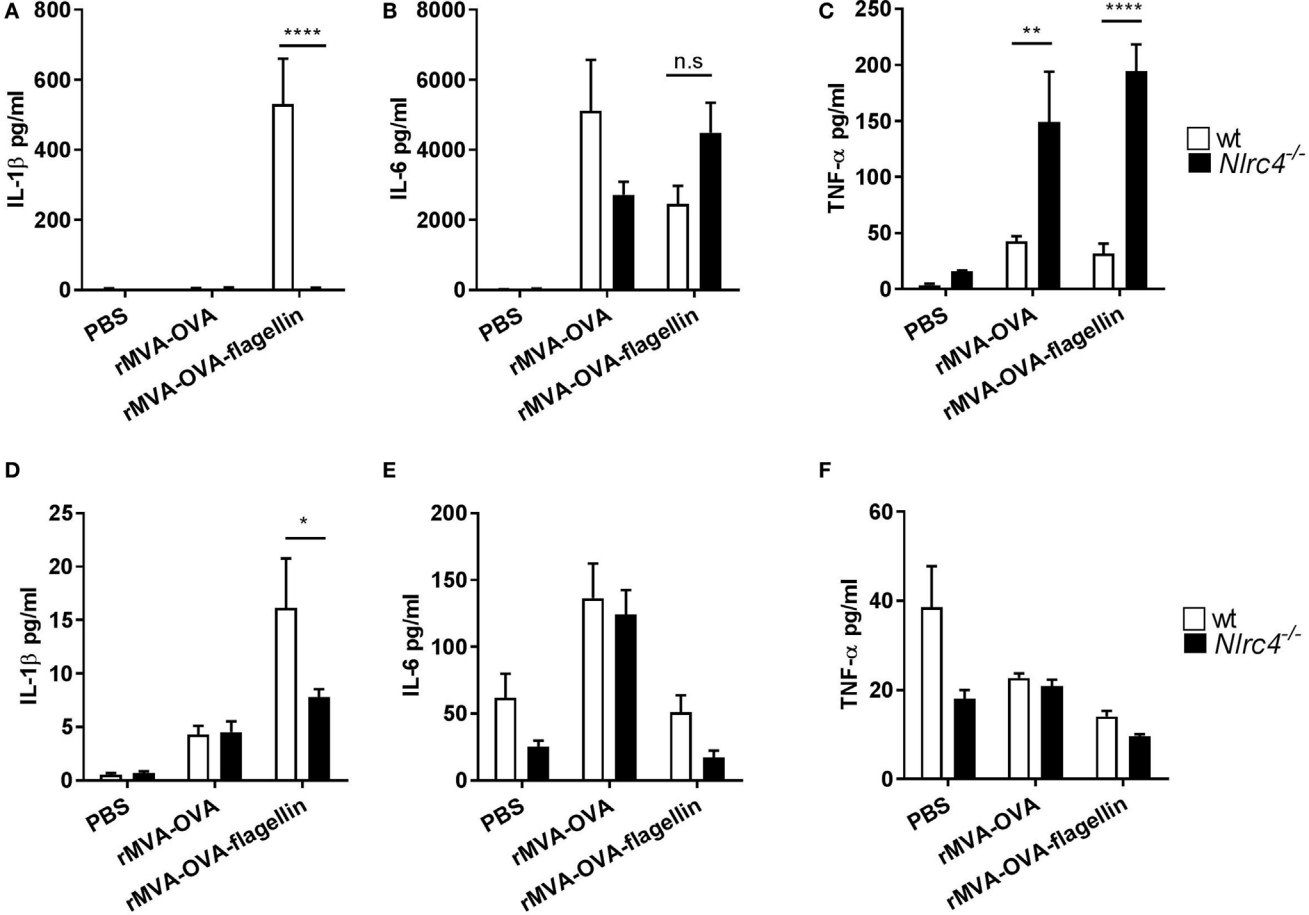
*In vivo* lung IL-1β is mediated by NLRC4. WT and *Nlrc4^−/−^* mice were immunized *i.n*. with rMVA vaccine, also encoding OVA (rMVA-OVA), rMVA-OVA-flagellin or PBS. **(A–C)** bronchoalveolar was collected 24 h later and IL-1β **(A)**, IL-6 **(B)** and TNF-α **(C)** were measured with Luminex. **(D–F)** *Ex vivo* overnight culture of lungs cells from wt and *Nlrc4^−/−^* immunized mice. The supernatant was collected and IL-1β **(D)**, IL-6 **(E)**, and TNF-α **(F)** were measured with Luminex. Statistical analyses were done by two-way ANOVA: *p < 0.05, **p < 0.005, ****p < 0.0001.

We further elucidated the role of NLRC4 activation by rMVA-OVA-flagellin *in vivo*. Wt and *Nlrc4^−/−^* mice were *i.n*. immunized and IL-1β was significantly produced in the BAL of wt mice, while abrogated in *Nlrc4^−/−^* immunized mice (Figure 4A). No loss of neither IL-6 (Figure 4B) nor TNF-α (Figure 4C) was observed in the BAL of rMVA-OVA-flagellin *Nlrc4^−/−^* immunized mice. Interestingly, higher TNF-α levels were observed in *Nlrc4^−/−^* mice compared to wt, which would indicate a negative role of the NRLC4 inflammasome on the NF-κb-mediated pathway of TNF-α production in our model.

Furthermore, the role of NLRC4 in *ex vivo* isolated lung cells was also investigated (Figures 4D–F). Lung cells from wt or *Nlrc4^−/−^* immunized mice were cultured *ex vivo*, and IL-1β, IL6, and TNF-α were determined. Results show elevated IL-1β production from lung cells of wt mice in response to rMVA-OVA-flagellin-immunization, which was reduced in that of *Nlrc4^−/−^* mice (Figure 4D). In contrast, no significant changes in IL-6 (Figure 4E) or TNF-α (Figure 4F) were observed.

Together, these results show that rMVA-OVA-flagellin recognition in the lung and IL-1β production is indeed dependent on the NLCR4 inflammasome.

### Mucosal Application of rMVA-Flagellin Vaccine Enhances CD8^+^ T Cell Responses

rMVA vaccine, also encoding OVA-flagellin leads to enhancement of innate immune recognition after *i.n*. application. Therefore, we addressed whether adaptive immune responses would also be influenced by this adjuvant effect *in vivo*. Our vaccination schedule consists of an *i.n*. prime with either rMVA-OVA or rMVA-OVA-flagellin, followed by a homologous boost. At day 35, antigen-specific CD8 responses at systemic and mucosal sites were assessed.

**Figure 5:**
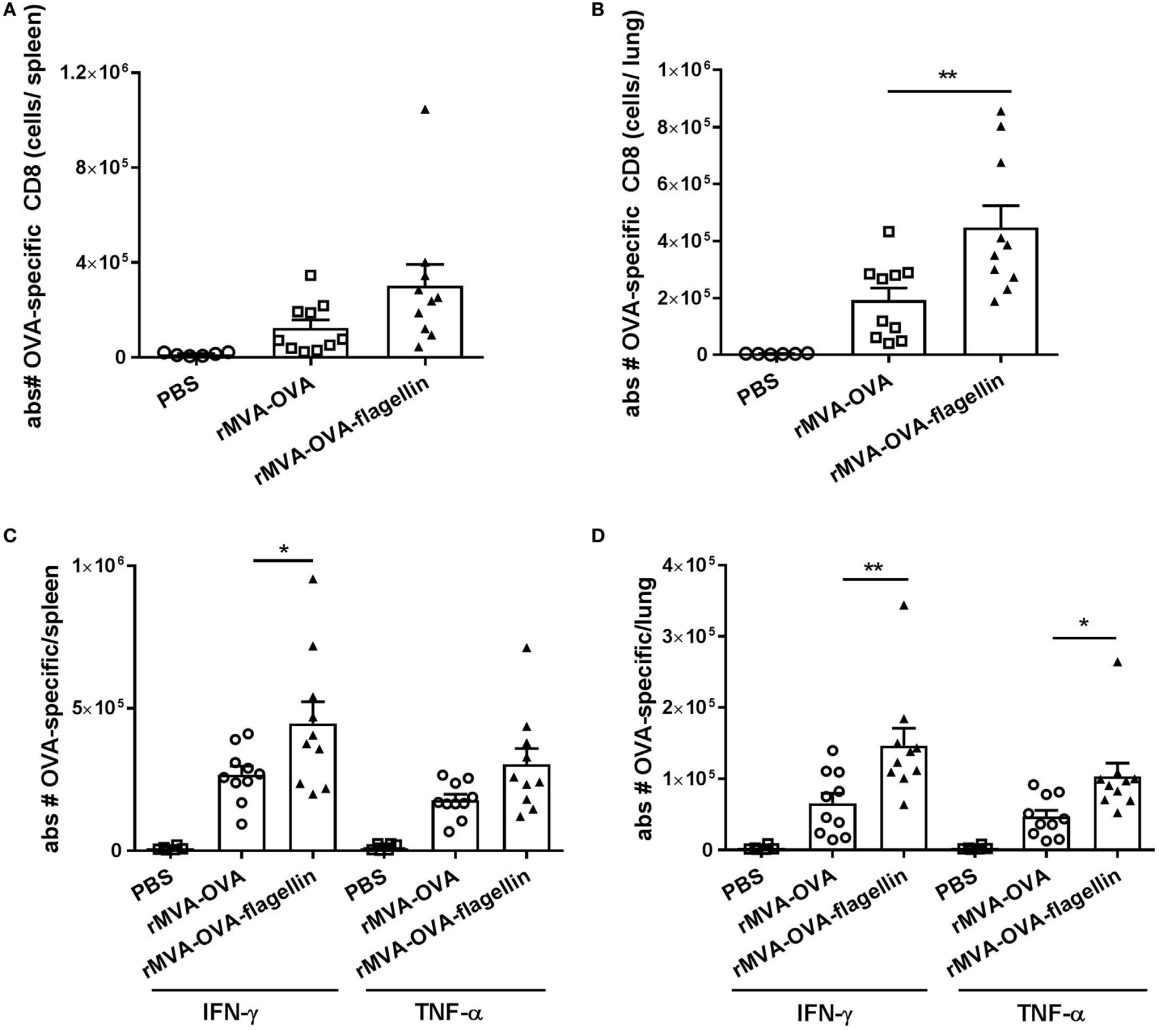
rMVA vaccine, also encoding OVA (rMVA-OVA)-flagellin enhances antigen-specific and cytokine-producing memory CD8 responses in the spleen and lung. Mice were immunized *i.n*. with rMVA-OVA, rMVA-OVA-flagellin or PBS. **(A,B)** OVA peptide-specific dextramer positive cells were determined in the spleen **(A)** and lungs **(B)**. Spleen and lung cells were stained for activated memory CTL and stained as CD44^+^OVA^+^ cells gated on CD8^+^ lymphocytes after live/dead staining. **(C)** Spleen and **(D)** lung cells were stained for activated cytokine-producing memory CTL as CD44^+^CD8^+^IFN-γ^+^TNF-α^+^ cells. Results represent the mean (±SEM) of individual mice (10 mice) combined from two independent experiments. Statistical analyses were done by one-way ANOVA: *p < 0.05, **p < 0.005.

In the spleen, no increase in absolute numbers of OVA peptide-specific dextramer positive cells (Figure 5A) between *i.n*. rMVA-OVA-flagellin and rMVA-OVA-immunized mice was observed (rMVA: 125,000 ± 34,000, rMVA-flagellin: 302,000 ± 90,000). Distinctively, in the lungs (Figure 5B), the data show a significant increase in OVA peptide-specific dextramer positive cells following rMVA-OVA-flagellin immunization (Figure 5B) (rMVA: 191,000 ± 420,004, rMVA-flagellin: 47,000 ± 76,000).

The effector functions of memory CD8^+^ T cells, known to rapidly produce IFN-γ and TNF-α (24) following *in vitro* restimulation with OVA peptide was assessed. In the spleen, no significant difference in the number of TNF-α^+^ OVA-specific CD8 T cells after rMVA-OVA-flagellin immunization could be observed (Figure 5C) (rMVA-flagellin: 303,000 ± 55,000, rMVA: 178,000 ± 20,000). In contrast, an increase in IFN-γ^+^ OVA-specific CD8 T cells ocurred (rMVA-flagellin: 447,000 ± 75,000, rMVA: 267,000 ± 30,000) (Figure 5C).

In the lungs, a significantly increased number of cytokine-producing OVA-specific CD8 T cells was observed in rMVA-OVA-flagellin-immunized mice (Figure 5D) (rMVA-flagellin: 147,000 ± 24,000 IFN-γ^+^, 103,500 ± 18,000 TNF-α^+^ OVA-specific CTLs; rMVA: 66,000 ± 14,000 IFN-γ^+^ and 47,000 ± 9,000 TNF-α^+^ OVA-specific CTLs).

Altogether, these data demonstrate the adjuvant-enhanced adaptive immunity of rMVA-OVA-flagellin, characterized by an increased neo-antigen-specific CD8 response and frequency of cytokine-producing memory CTLs. This significant enhanced antigen-specific immune response occurs in the lungs, at the site of immunization, with a similar trend observed in the spleen.

### Induction of GI Immune Responses by *i.n*. rMVA Immunization

In recent years, *i.n*. immunization was described to elicit both upper-respiratory, genital, and GI tract immune responses (21, 22, 25, 26). Therefore, we determined if rMVA-OVA and rMVA-OVA-flagellin administered *i.n*. could also induce immune responses in the GI tract.

**Figure 6:**
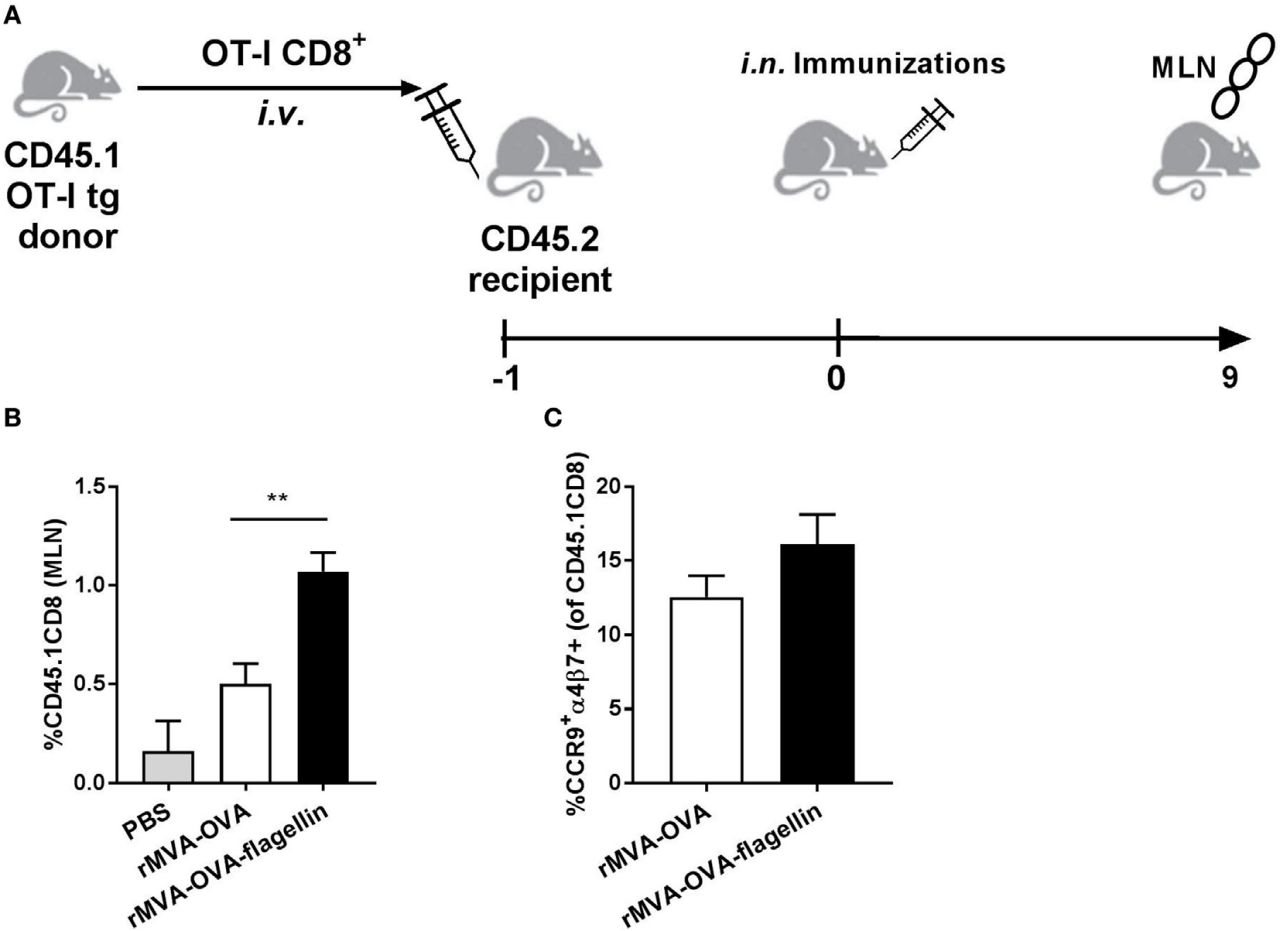
Increased transferred OT-I CD8^+^ T cells to the mesenteric lymph nodes (MLN) of *i.n*. rMVA vaccine, also encoding OVA (rMVA-OVA)-immunized mice. **(A)** Mice were transferred *i.v*. with donor CD45.1^+^OT-I CD8^+^ T cells and a day later immunized *i.n*. with rMVA-OVA, rMVA-OVA-flagellin, or PBS. **(B)** 9 days after immunization, MLN were stained for CD45.1^+^CD8^+^. The number of CD45.1^+^ CD8^+^ T cells/MLN was determined. **(C)** Gut-homing receptors, α4β7 and CCR9, on transferred OT-I CD8^+^ T cells were analyzed. The number of α4β7^+^CCR9^+^CD45.1^+^CD8^+^ T cells/MLN was determined. Results represent the mean (±SEM) of individual mice from two pooled independent experiments. Statistical analyses were done by one-way ANOVA: **p < 0.005.

In order to increase the number of CD8 T cell precursors, we used an adoptive transfer of OVA-specific CD8^+^ T cells from donor CD45.1^+^ OT-I mice into C57BL6 wt recipient mice (Figure 6A), followed a day later by *i.n*. rMVA-OVA and rMVA-OVA-flagellin immunization. When mesenteric lymph nodes (MLN) were isolated 9 days after immunization, an increase of OT-I CD8^+^ T cells in the MLN was observed (Figure 6B). More specifically, a twofold increase in OT-I CD8^+^ T cells could be detected in rMVA-OVA-flagellin-immunized mice, compared to rMVA-OVA-immunized mice (rMVA: 0.5% ± 0.1, rMVA-flagellin: 1% ± 0.09) (Figure 6B).

These OT-I CD8^+^ T cells also expressed the gut-homing receptors α4β7 and CCR9 (Figure 6C).

Altogether, the increase in OT-I CD8^+^ T cells in the MLN indicates that *i.n*. MVA-OVA is associated with a CD8 response in the GI tract, which is enhanced when using rMVA-OVA-flagellin.

### *i.n*. Administration of rMVA-Flagellin Induces Stronger Serum Antibody and Mucosal IgA Responses

Having previously described that an *i.n*. rMVA-OVA-flagellin vaccine induced stronger adaptive T cell responses, we also wanted to determine the contribution of rMVA-OVA-flagellin on systemic and mucosal humoral immune responses.

**Figure 7:**
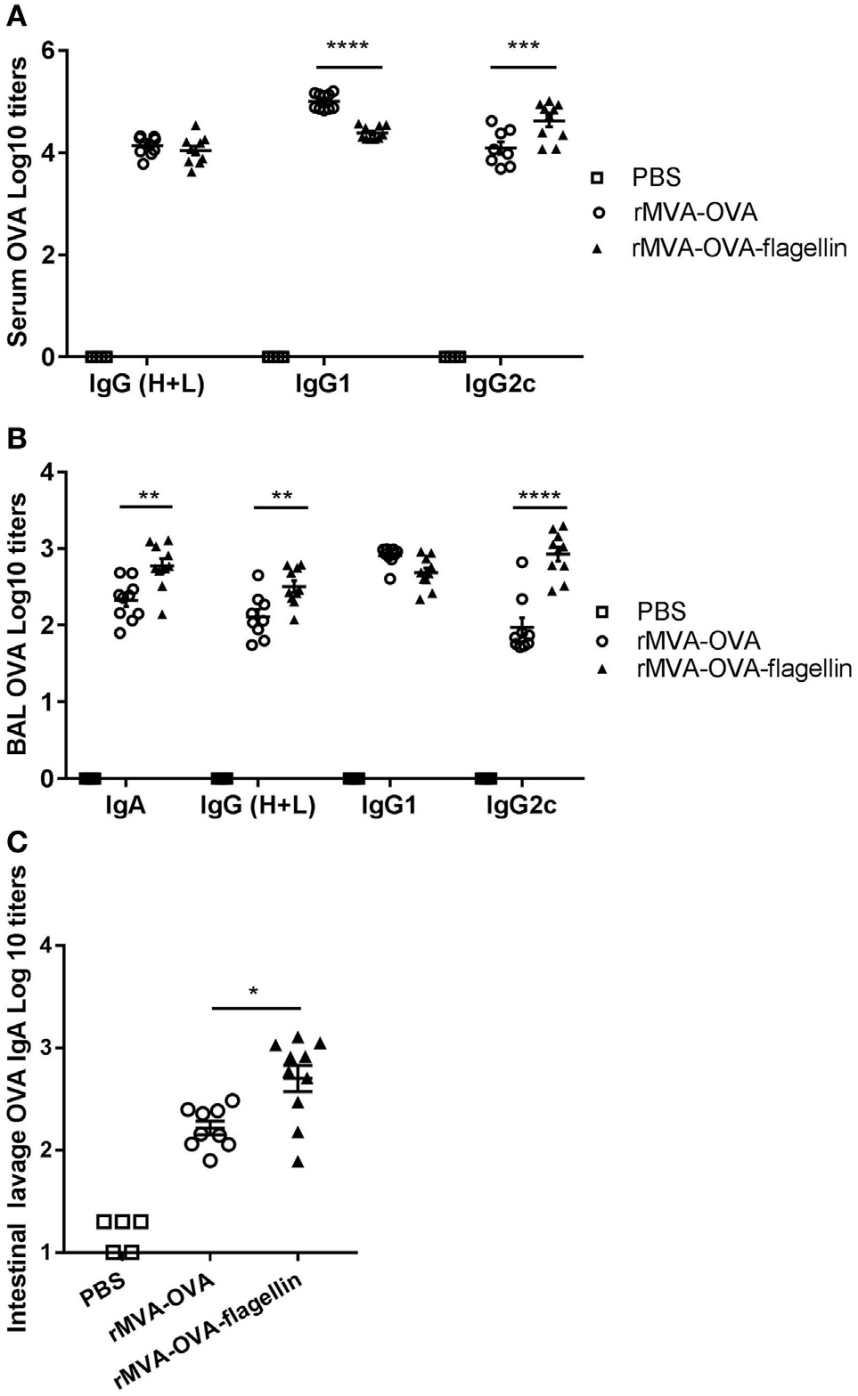
rMVA vaccine, also encoding OVA (rMVA-OVA)-flagellin induces OVA-specific IgG2c antibody responses in the serum, bronchoalveolar (BAL), and mucosal IgA. Mice were immunized *i.n*. with rMVA-OVA, rMVA-OVA-flagellin, or PBS. **(A)** Serum, **(B)** BAL, and **(C)** intestinal lavages were collected at day 35 and analyzed for IgG(H + L), IgG1, IgG2c, and IgA anti-OVA responses by ELISA. Data are shown as individual mice (8–10 mice) combined from two independent experiments with bars representing the mean (±SEM). Statistical analyses were done by one-way ANOVA **(C)**: *p < 0.05, or two-way ANOVA **(A,B)** **p < 0.005, ***p < 0.0005, ****p < 0.0001.

Serum OVA-specific IgG was measured. Our data show no significant difference in OVA-specific total IgG titers between rMVA-OVA-flagellin and rMVA-OVA-immunized mice (Figure 7A). In order to identify the type of immune response generated, specific IgG isotype responses were measured. Whereas higher serum OVA-specific IgG1 titers were observed in rMVA-OVA-immunized mice (Figure 7A), enhanced OVA-specific IgG2c titers (Figure 7A) were observed after rMVA-OVA-flagellin immunization, suggesting a stronger bias toward a Th1 immune response.

Bronchoalveolar OVA-specific IgA antibody responses were determined and significant enhanced mucosal IgA responses were observed in rMVA-OVA-flagellin-immunized mice (Figure 7B). Furthermore, an increase in BAL anti-OVA-IgG titers, together with enhanced BAL IgG2c antibody responses (Figure 7B) was also observed in those mice, suggesting again a bias toward a Th1 immune response. OVA-specific IgA could also be detected in the LP of immunized mice (Figure 7C) with a clear increase in IgA titers following *i.n*. rMVA-OVA-flagellin immunization (Figure 7C).

Altogether these data suggest that *i.n*. rMVA-OVA-flagellin immunization leads to improved serum antibody responses and enhanced mucosal IgA in the upper-respiratory and GI tracts.

### Reduced Adaptive Immunity in *Nlrc4^−/−^* Mice

Having described the contribution of NLRC4 in rMVA-OVA-flagellin-specific enhanced innate immune responses, we wanted to determine the requirement of NLRC4 for the adjuvant effect observed in *i.n*. rMVA-OVA-flagellin vaccinated mice.

**Figure 8:**
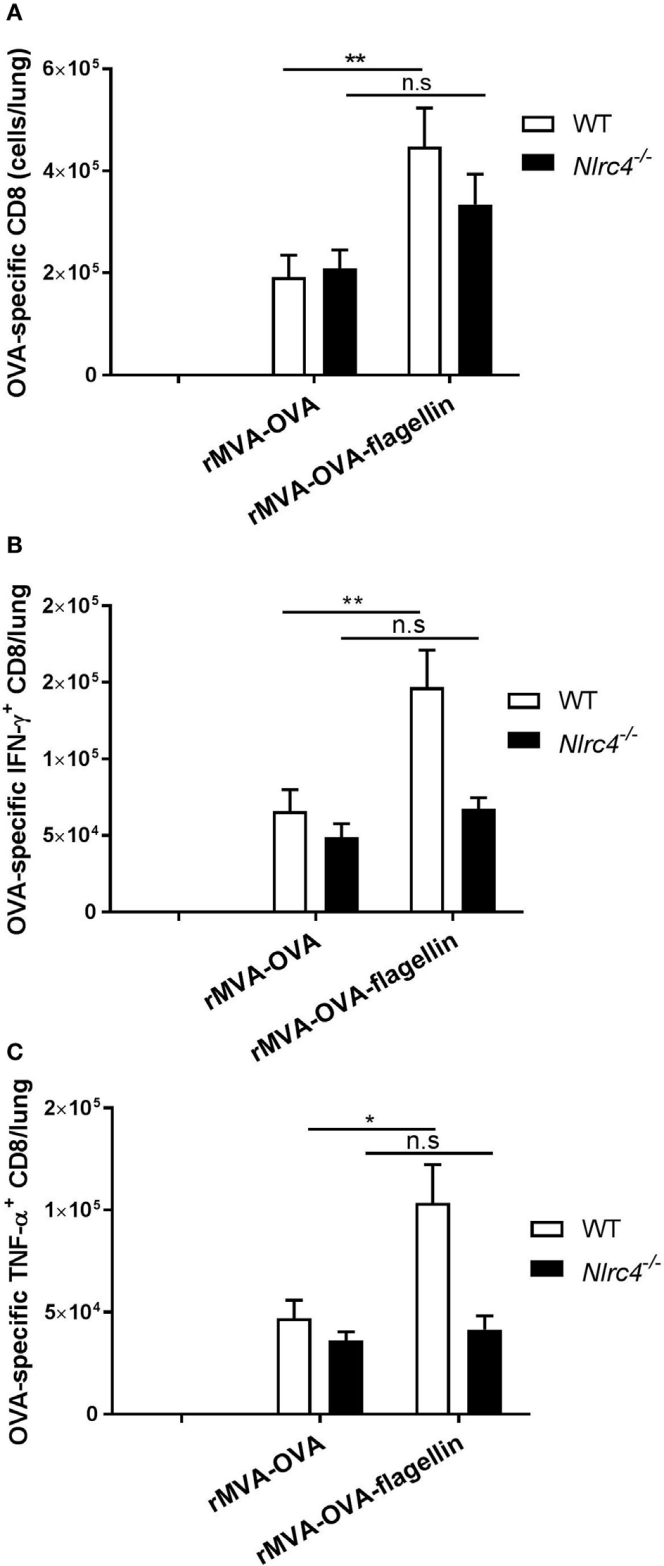
*Nlrc4^−/−^* mice exhibit reduced adaptive immune responses. WT and *Nlrc4^−/−^* mice were immunized *i.n*. with rMVA vaccine, also encoding OVA (rMVA-OVA), rMVA-OVA-flagellin or PBS. **(A)** Lung OVA-specific CTL were determined following staining of lung memory CTL, as CD44^+^CD8^+^OVA^+^ cells. **(B,C)** Lung cells were stained for activated cytokine-producing memory CTL as CD44^+^CD8^+^IFN-γ^+^TNF-α^+^ cells. **(B)** IFN-γ-producing CTL and **(C)** TNF-α-producing CTL/lungs were determined. Results represent the mean (±SEM) of individual mice (10 mice) combined from two independent experiments. Statistical analyses were done two-way ANOVA: *p < 0.05, **p < 0.005, n.s., non-significant.

*Nlrc4^−/−^* mice were immunized and adaptive CD8 T cell responses in the lung determined. Our results show that compared to wt mice, a lack of enhanced OVA-specific CD8 responses could be observed in *i.n*. rMVA-OVA-flagellin-immunized *Nlrc4^−/−^* mice (Figure 8A).

Moreover, we also determined the number of cytokine-producing OVA-specific CTLs in rMVA-OVA-flagellin-immunized *Nlrc4^−/−^* mice (Figures 8B,C). Convincingly, our results show that the enhancement of cytokine-producing CTLs observed in wt mice does not occur in rMVA-OVA-flagellin-immunized *Nlrc4^−/−^* mice, with similar numbers of IFN-γ^+^ and TNF-α^+^ OVA-specific CTLs as that of rMVA-OVA-immunized mice.

This suggests that NLRC4 plays a preferential role in the rMVA-OVA-flagellin-induced lung CD8 T cell responses following *i.n*. immunization.

### NLRC4-Dependent IgG2c and Mucosal IgA Responses following rMVA-Flagellin-Immunization

The enhanced T cell responses in the lung of rMVA-OVA-flagellin-immunized mice were largely dependent on functional NLRC4. Accordingly, we wanted to determine the role of NRLC4 in rMVA-OVA-flagellin-induced humoral responses.

**Figure 9:**
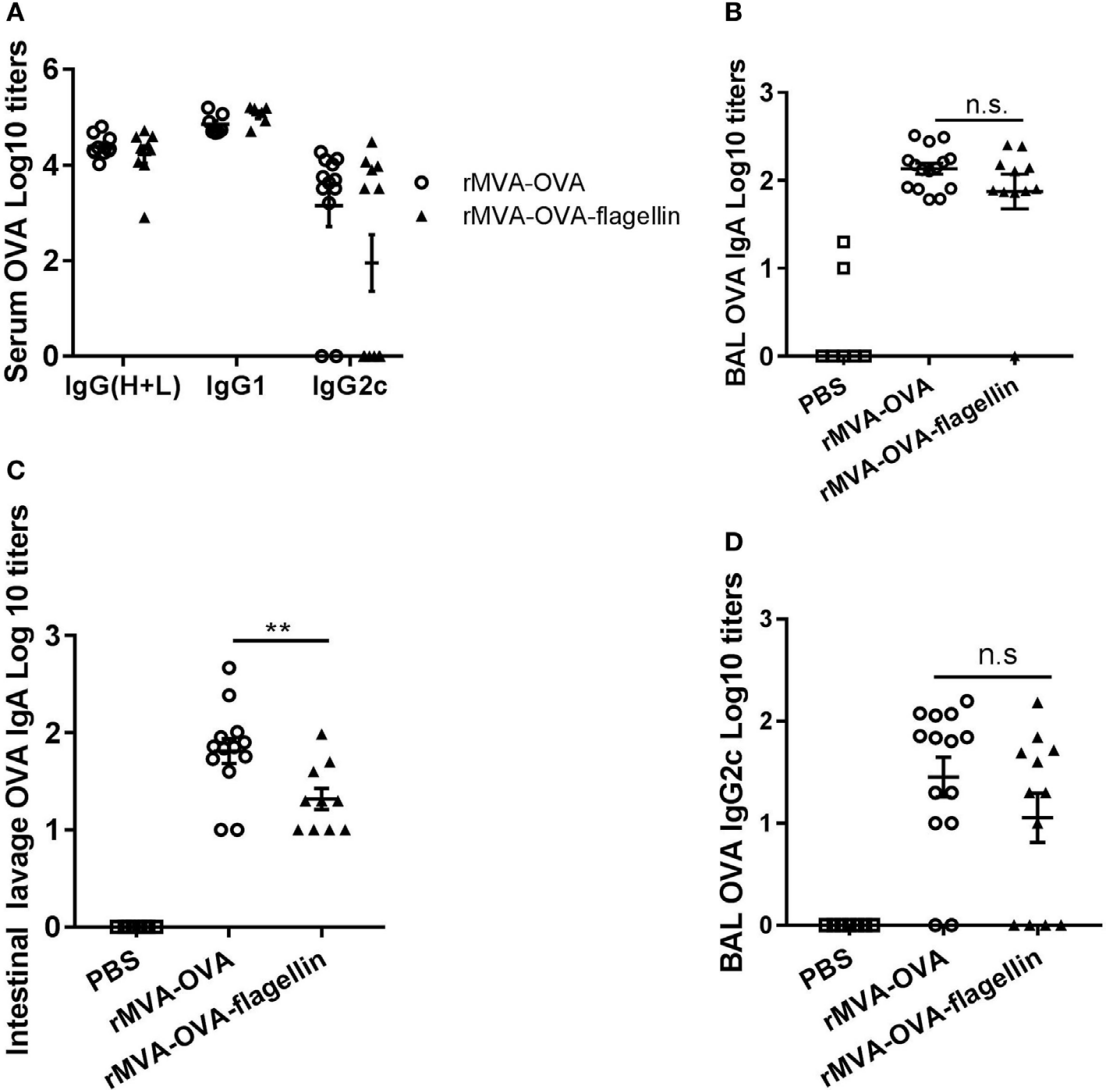
Reduced serum, mucosal IgG2c, and IgA antibody responses in *Nlrc4^−/−^* mice during rMVA vaccine, also encoding OVA (rMVA-OVA)-flagellin immunization. *Nlrc4^−/−^* mice were immunized *i.n*. with rMVA-OVA, rMVA-OVA-flagellin, or PBS. Serum **(A)**, bronchoalveolar (BAL) **(B,D)** and intestinal lavages **(C)** were taken at day 35, and analyzed for IgG(H + L), IgG1, IgG2c, and IgA anti-OVA responses by ELISA. **(A)** Serum anti-OVA IgG(H + L), IgG1, and IgG2c responses. **(B)** BAL anti-OVA IgA. **(C)** Intestinal lavage anti-OVA IgA **(C)** and **(D)** BAL anti-OVA IgG2c responses. Data are shown as individual mice (10–14 mice) combined from three independent experiments with bars representing the mean (±SEM). Statistical analyses were done by one-way ANOVA: **p < 0.005, ns non-significant.

First, we determined total serum IgG titers. Similar to wt mice (Figure 7A), there was no difference in total IgG titers between rMVA-OVA and rMVA-OVA-flagellin-immunized *Nlrc4^−/−^* mice (Figure 9A). In contrast, striking differences were observed with regard to IgG2c responses. While rMVA-OVA-flagellin-immunized wt mice showed distinct enhanced serum OVA-specific IgG2c titers (Figure 7A), this effect was completely abrogated in *Nlrc4^−/−^* mice (Figure 9A), suggesting an indispensable role of NLRC4 in rMVA-OVA-flagellin-induced enhanced Th1 responses.

Having observed this striking phenotype, we determined whether mucosal humoral responses might also be impaired in the absence of NLRC4. Our results show that the increased BAL IgA response of rMVA-OVA-flagellin-immunized wt mice (Figure 7B) is also abrogated in *Nlrc4^−/−^* mice (Figure 9B), with similar titers of OVA-specific IgA observed between rMVA-OVA and rMVA-OVA-flagellin-immunized *Nlrc4^−/−^* mice (Figure 9B). Comparable findings were observed in intestinal lavages of *Nlrc4^−/−^* mice (Figure 9C), whereby enhanced IgA responses were also abrogated compared to wt mice (Figure 7C). Thus, this result suggests another essential role of NLRC4 in rMVA-OVA-flagellin-induced mucosal IgA responses.

Since rMVA-OVA-flagellin induced enhanced bias in mucosal Th1 responses in wt mice (Figure 7B), we also addressed the role of NLRC4 in rMVA-OVA-flagellin-specific induced mucosal IgG2c antibody responses. Quite strikingly, a loss of rMVA-OVA-flagellin-induced IgG2c responses was observed in the BAL of *Nlrc4^−/−^* (Figure 9D) compared to wt mice (Figure 7A). This reduction of IgG2c strongly suggests that NLRC4 signaling is a prerequisite for rMVA-OVA-flagellin-induced enhanced IgG2c antibody responses.

Altogether these data point toward a role of NLRC4 in rMVA-flagellin-induced Th1-biased humoral systemic and mucosal immune responses.

## Discussion

In this study, we assessed immunogenicity of a novel rMVA-flagellin vaccine upon mucosal application. Notably, mice immunized *i.n*. with this vaccine exhibited enhanced innate and adaptive mucosal immune responses. Moreover, we were able to show an essential role of NLRC4 in flagellin-enhanced immunogenicity and the essential requirement of NLRC4 for flagellin-induced IgG2c humoral and mucosal IgA responses.

The *in vivo* analysis of mucosal innate immune responses after *i.n*. rMVA-flagellin immunization revealed the production of IL-1β in the lungs suggesting inflammasome-dependent activation. Recent literature described the NAIP family of NLR proteins providing specificity to the activation of NLRC4 by bacterial ligands (27). Employing NLRC4-deficient mice and a NLRC4-expressing human macrophage cell line, we clearly could show the essential role of NLRC4 for rMVA-flagellin-induced innate immune responses. In addition to IL-1β, we could detect enhanced levels of TNF-α, suggesting that rMVA-flagellin activates both flagellin-specific innate signaling pathways, NLRC4 and TLR5. Interestingly, IL-18 was detected, albeit without any increase in rMVA-flagellin-immunized mice, suggesting that IL-18 is mainly induced by the MVA backbone and not further increased by flagellin. Notably, it is the NLRC4 and TLR5 mucosal tissue-specific expression on innate immune cells and epithelial cells of the lungs and in the intestines (10, 28), which prompted us to investigate *i.n*. application of our vaccine and determine if mucosal innate cells were poised to respond to rMVA-flagellin, and so far innate immune responses corroborate this hypothesis.

Having observed flagellin-specific NLRC4-dependent IL-1β in the lungs, the source of IL-1β, was investigated. Most of the inflammasome research has focused on macrophages and DCs (29, 30), shown to be an important, but not the sole source of IL-1β during rMVA-flagellin vaccination. Following investigation in *Flt3-L^−/−^* and CD11c-DTR mice, IL-1β response was significantly reduced, although never completely abrogated. However, these two mouse models exhibit caveats, which might explain this finding. In *Flt3-L^−/−^* mice, known to harbor drastically reduced numbers of dendritic cells (31), macrophages remain present, whereas in CD11c-DTR mice, a proportion, but not all of the different macrophage populations and NK cells might be depleted (32). Therefore, a role of macrophages cannot be excluded for the remaining production of IL-1β, since the human monocytic THP-1 cells also produce IL-1β after rMVA-flagellin stimulation. Therefore, it supports the role of a DC/myeloid population as the innate source of IL-1β. In contrast, IL-6, TNF-α, and IL-18 levels were not affected in the BAL CD11c-DTR mice, as well as marginally reduced in the BAL of *Flt3-L^−/−^* mice suggesting for a role of a non-DC cell population. *In vitro* FL-DCs stimulated with rMVA-flagellin also demonstrate both NLRC4 and TLR-driven cytokine production, with IL-1β, IL-18, and IL-6 and TNF-α observed, respectively, simultaneously largely lost in rMVA-flagellin-stimulated *Nlrc4^−/−^* and *Myd88^−/−^* FL-DCs. In order to further confirm rMVA-flagellin-specific inflammasome activation in DCs, the role of caspase-1, required for the cleavage of pro- IL-1β and pro-IL-18 into active IL-1β and IL-18 (5), was investigated *in vitro*. Using a caspase-1 inhibitor, abrogation of IL-1β was observed in rMVA-flagellin-stimulated FL-DCs, suggesting a NLRC4-dependent caspase-1 activation in DCs.

Moreover, we show that our findings are also relevant in human cells. IL-1β and IL-6 are induced in rMVA-flagellin-stimulated THP-1 cells, as a result of TLR5 expression from those cells. THP-1-overexpressing NLRC4 stimulated with rMVA-flagellin, exhibit an even greater IL-1β production, as well as IL-6, TNF-α and to a certain extent IL-18. Hence, this further supports the potential translation and application of rMVA-flagellin to a human setting. Flagellin is indeed being investigated, as a fusion vaccine, already in clinical development for the treatment of Influenza virus (33) and evaluated in human experimental studies of breast cancer using human breast cancer cell lines (34).

Given the ability of rMVA-flagellin to induce enhanced innate immune responses and in order to translate our innate findings into an applied vaccination setting, we tested rMVA-flagellin in a classical prime-boost vaccination model. *I.n*. rMVA-flagellin immunization show enhanced lung memory CTL responses and to a lesser extent in the spleen. We also demonstrate the ability of rMVA-flagellin to increase the number of highly functional memory CD8^+^ T cells, especially in the respiratory tract. Serum and BAL humoral responses were also investigated. Interestingly, although no difference in total serum IgG titers between rMVA and rMVA-flagellin-immunized mice was observed, a distinct increase in the IgG2c isotype was detected, indicating a more pronounced Th1 immune response. Notably, in the BAL, both IgG and IgG2c antibody responses were increased. These data point to an overall beneficial effect of rMVA-flagellin in promoting humoral immune responses.

The strategy using *i.n*. application for rMVA-flagellin was to strengthen mucosal IgA responses. Accordingly, BAL IgA responses were significantly increased when rMVA-flagellin was used. In light of these findings, the rMVA-flagellin-specific adjuvant effect on innate and adaptive immune responses makes rMVA-flagellin a promising candidate for a mucosal vaccine.

Having established rMVA-flagellin as a vaccine with distinct adjuvant properties, we wanted to address the mechanism responsible for rMVA-flagellin-enhanced immunogenicity. The *in vivo* role of NLRC4 in rMVA-flagellin-induced lung innate immune responses was clearly demonstrated. To this end, we further explored the role of the NLRC4 *in vivo*, and determined if the latter was a critical component in rMVA-flagellin-enhanced adaptive immune responses. A partial loss of the enhanced OVA-specific CD8^+^ T cell responses was observed in the lungs of *Nlrc4^−/−^* mice. Notably, the effect of rMVA-flagellin on lung memory CTL response was reduced though still present. Nevertheless, a reduction in IFN-γ and TNF-α-producing lung CTLs was observed in *Nlrc4^−/−^* mice. Therefore, the CD8 T cell responses specifically enhanced by rMVA-flagellin appear to be dependent on NLRC4 inflammasome activation. We further addressed the contribution of the NLRC4 inflammasome in rMVA-flagellin-induced humoral responses. Notably, striking differences were observed between wt and *Nlrc4^−/−^* mice. Whereas rMVA-flagellin-immunized wt mice exhibit enhanced serum IgG2c titers, this increase was completely abolished in *Nlrc4^−/−^* mice. Similarly, in the BAL, the absence of NLRC4 signaling lead to the loss of the enhanced rMVA-flagellin-induced IgG2c response observed in wt mice. Likewise, enhanced mucosal IgA responses from the BAL and intestinal lavages were completely absent in rMVA-flagellin-immunized *Nlrc4^−/−^* mice, supporting a NLRC4-enhanced production of IgA in the LP. Collectively, these data indicate a critical role of NLRC4 in rMVA-flagellin-induced adaptive immunity, specifically humoral IgG2c, and mucosal IgA responses. This finding highlights the importance of using an *i.n*. route for rMVA-flagellin for the development of vaccines against enteric pathogens.

To a certain extent, using *Nlrc4^−/−^* mice enabled us to also ascertain the contribution of TLR5 in rMVA-flagellin adjuvanticity after *i.n*. immunization. We can thus speculate that the flagellin-specific adjuvant effect seen in rMVA-flagellin relies on NLRC4, while TLR5 signaling alone is neither sufficient nor required for rMVA-flagellin’s immunogenicity in the absence of NLRC4. Indeed, TLR5 signaling does not seem to compensate for NLRC4 deficiency, as the rMVA-flagellin-specific adjuvant effect is not sustained. Further investigations in *Tlr5^−/−^* mice would be necessary in order to demonstrate the contribution of TLR5 and would also allow us to assess whether in the absence of TLR5 signaling, NLRC4 has a dispensable role.

Intranasal application has proven to be a very attractive route of immunization, due its benefits in eliciting immune responses in the urogenital gastro-intestinal (UGI) tract. Therefore, we were interested to further explore the potential advantage of rMVA-flagellin, in inducing CD8 response immune responses in the GI tract after *i.n*. application. Following adoptive transfer of CD45.1^+^ OT-I CD8^+^ T cells prior to *i.n*. rMVA-flagellin immunization, enhanced OT-I CD8^+^ T cells to the MLN was observed. These migrated CD8^+^ T cells upregulated expression of the gut-homing receptors α4β7 and CCR9, suggesting a better ability of rMVA-flagellin to confer intestinal tissue tropism to CD8^+^ T cells. Therefore, our finding would also support *i.n*. rMVA-flagellin immunization-induced priming of lung DCs and migration of primed T cells to the MLN. Moreover, mucosal IgA responses could be detected in the LP of both rMVA and rMVA-flagellin-immunized mice, indicating establishment of GI humoral responses. Notably, higher IgA levels were observed in rMVA-flagellin immunized mice, highlighting the advantage of the *i.n*. route for enhanced mucosal immunogenicity and GI specificity. Collectively, these results suggest that *i.n*. application of rMVA vaccines can elicit the development of GI immune responses.

In summary, our data indicate the beneficial role of incorporating flagellin into rMVA. rMVA-flagellin possesses the ability to enhance both innate and adaptive immune responses and thus improves the vaccine CD8 and antibody response. More specifically, using an *i.n*. route of vaccination enables the development of mucosal and GI immune responses. Moreover, the NLRC4 inflammasome appears to play an essential role in rMVA-flagellin-enhanced vaccine response. Collectively, this study shows great promise for the development of a flagellin-adjuvanted rMVA vaccine with mucosal application for the development of vaccines against a wider range of pathogens, targeting disease of both the upper-respiratory and the UGI-tract.

## Materials and Methods

### Mice

C57BL/6J (H-2^b^) mice were purchased from Janvier Labs. C57BL/6-Tg (TcraTcrb) 1100MjbJ (OT-I), and B6.SJL-Ptprca Pepcb/BoyJ (CD45.1) mice were obtained from the University of Zurich and bred to obtain CD45.1^+^ OT-I mice, respectively. *CD11c*-DTR, *Myd88^−/−^, Nlrc4^−/−^*, and *Flt3-L^−/−^* mice were obtained from the University of Zurich. Mice were bred and maintained either in the animal facilities at Bavarian Nordic GmbH or at the University of Zurich according to institutional guidelines.

### Generation of MVA-BN Recombinants

All recombinant virus vectors used for this study were based on a cloned version of MVA-BN^®^ in a bacterial artificial chromosome (BAC). The generation of MVA recombinants was carried out as described recently (24, 34). MVA-BN^®^ was developed by Bavarian Nordic and is deposited at the European Collection of Cell Cultures (ECACC) (V00083008). Briefly, the The pS promoter was cloned upstream of the open-reading frame for chicken OVA. The pHyb promoter was developed and described by Baur et al. (35) and comprises a late element from the promoter directing the expression of the AT1 protein in cowpox virus and five tandemly arranged early elements derived from a modified p7.5 promoter. The pHyb promoter was cloned upstream of the open-reading frame for *Salmonella typhimurium* FliC, hereafter referred to as Flagellin. Infectious viruses were reconstituted from BACs by transfecting BAC DNA into BHK-21 cells and superinfecting them with Shope fibroma virus as a helper virus. After three additional passages on primary chicken embryo fibroblasts, helper virus free MVA-OVA, MVA-OVA-flagellin viruses were obtained. All viruses used in animal experiments were purified twice through a sucrose cushion.

### Immunizations

For intranasal immunizations, mice were anesthetized and immunized with a total volume of 50–60 µl containing 3.5 × 10^7^ TCID_50_ of the various MVA recombinants. Mice received two immunizations on days 0 and day 21 with 3 weeks interval. Immune responses were evaluated 2 weeks after the final immunization at day 35. Serum, BAL, and intestinal lavages were taken on the final day for antibody titers. Groups of five mice were used.

### *In Vivo* DC Cell Depletion

For lung DC depletion, *CD11c*-DTR mice were treated *i.n*. with 100 ng diphtheria toxin (DT; Sigma-Aldrich) in 50 µl a day prior to immunizations.

### Adoptive Cell Transfer

CD45.1^+^ OT-I mice were used as cell donors for adoptive cell transfer into syngeneic CD45.2^+^ C57BL/6J recipient animals. Lymphocytes were isolated from spleen and OT-I CD8^+^ T cells purified using EasySep kits from StemCell Technology according to the manufacturer’s protocol. 1 × 10^4^ OT-I CD8^+^ T cells were injected intravenously *via* the tail vein in a 100-µl volume a day prior to *i.n*. immunization.

### Cell Isolation (Spleen, Lung, MLN)

Spleen and lung cells were harvested from mice and incubated with 1 mg/ml collagenase/DNase in RPMI 1640 media containing 2% FCS for 30 min at 37°C on a shaker. Single-cell suspensions were then prepared by mechanically disrupting the organs through a 70-µm filter, washed, and counted. Spleen cells were then subjected to red blood cell lysis (Erylysis, Sigma-Aldrich), whereas lung cells were isolated by centrifugation with 44% Percoll, then washed and counted.

Mesenteric lymph nodes were harvested in RPMI 1640 media containing 2% FCS, disrupted mechanically through a 70-µm cell strainer. Mononuclear cells were washed and counted.

### Flow Cytometry

Spleen and lung lymphocytes were stained with PE-conjugated MHC class I H-2k^b^ dextramers loaded with OVA_257–264_-peptide (SIINFEKL) (Immudex), for the detection of OVA-specific CD8 T cells, respectively, followed by anti-CD8α, CD44, and CD4. Antibodies were purchased from BD Biosciences, eBioscience, or BioLegend. For detection of adoptively transferred OT-I CD8^+^ T cells and gut-homing receptors, MLN cells were stained with anti-CD8α, CD45.1, CD44, α4β7, and CCR9. For intracellular cytokine staining, spleen and lung mononuclear cell suspensions were incubated with 2.5 µg/ml of MHC class I restricted peptides (OVA_257–264_) (Genscript) for 5–6 h at 37°C in complete RPMI in the presence of 1 µg/ml GolgiPlug (BD Biosciences). Cells were stained as before with anti-CD8α, CD44, CD4, IFN-γ, and TNF-α. Intracellular cytokine staining of IFN-γ and TNF-α were performed after fixation/permeabilization (BD Cytofix/Cytoperm, BD Biosciences). For live/dead discrimination cells were stained before fixation (LIVE/DEAD fixable violet dead cell staining kit, Life Technologies). For analysis, all cells were gated on live CD8^+^ T lymphocytes following live/dead staining. All cells were acquired using a digital flow cytometer (LSR II, BD Biosciences) and data were analyzed with FlowJo software (Tree Star).

### ELISA

Serum OVA-specific IgGs were determined. For detection of OVA-specific IgA, the BAL fluid was collected by flushing the trachea three times with 1 ml of cold PBS/0.5 mM EDTA with a cannula, centrifuged to pellet the cells from the BAL fluid, and collected for ELISA. Intestinal washes were collected by flushing the distal part of the small intestine with 1 ml PBS containing trypsin inhibitor, 50 mM EDTA and 0.1% BSA. The solution was spun at 4,000 *g* for 15 mins at 4°C. ELISAs were performed by coating 96-well plates with 5 µg/ml OVA, followed by blocking with PBS containing 5% FCS/0.05% Tween20. IgG, IgG1, IgG2c, and IgA were detected using HRP-conjugated antibodies, followed by TMB substrate. Absorbance was measured at 450 nm. ELISA titers were determined using linear regression analysis and Log10 titers calculated.

### *In Vitro* Generation of FL-DCs and Stimulation

Bone marrow (BM)-derived DCs cultured in the presence of Flt3-L (FL) (FL-DCs) were prepared as follows. BM cells obtained from flushed femurs and tibias, cultured in the presence of human recombinant FL for 8 days. FL-DCs (5 × 10^5^ DC/well) were stimulated with 4 TCID_50_/cell of rMVA, rMVA-flagellin, or with 1 µg/ml recombinant flagellin from *S. typhimurium* (Invivogen). FL-DCs were pre-treated with the transfection reagent Lyovec (Invivogen) for 15 mins. FL-DCs were pre-treated for 1 h with the caspase-1 inhibitor VX-765 (Invivogen), followed by infection with 4 TCID_50_/cell of rMVA or rMVA-flagellin. Cells were incubated overnight and the supernatant was collected and measured for cytokine production using the Luminex.

### *Ex Vivo* Culture of Lung DCs

Lung cells from PBS, rMVA, or rMVA-flagellin immunized mice (wt or *Nlrc4^−/−^*), were harvested as described previously. A total of 5 × 10^5^ cells/well were cultured *ex vivo* with 10 ng/ml IL-4, GM-CSF and rat IFN-γ overnight in RPMI media containing 10% FCS. The supernatant was collected and measured for cytokines using the ProcartaPlex^®^ Multiplex Immunoassay (Affymetrix).

### Cell Lines

The human monocytic cell lines THP-1 and THP-1-NLRC4 (Invivogen), stably overexpressing NLRC4 and naturally expressing TLR5 (23) were cultured in RPMI containing 10% FCS. Cells were stimulated with 4 TCID_50_/cell of rMVA, rMVA-flagellin overnight. Viruses were heat-inactivated for 10mins in a 70°C water-bath, and THP-1 and THP-1-NLRC4 cells stimulated with 4 TCID_50_/cell of rMVA, rMVA-flagellin overnight. The supernatant was collected and measured for cytokine production using the Luminex.

### Cytokine Assay-Luminex

Bronchoalveolar and harvested supernatants were measured for cytokines using the ProcartaPlex^®^ Multiplex Immunoassay (Affymetrix) according to the manufacturer’s protocol. Data were acquired on a MAGPIX^®^ system (Invitrogen). Results were analyzed using the Masterplex 2010 software (Hitachi Solutions).

### Statistical Analysis

Statistical analysis using one-way or two-way ANOVA with multiple comparisons was performed using GraphPad Prism 7 software. *p* value < 0.05 was considered significant.

## Ethics Statement

All animal experiments were approved by the animal ethics committee of the government of Upper Bavaria (Regierung von Oberbayern, Sachgebiet 54, Tierschutz) and were carried out in accordance with the approved guidelines for animal experiments at Bavarian Nordic GmbH.

## Author Contributions

SS designed the study, performed experiments, analyzed the data, and wrote the manuscript. HL and HH designed the study and revised the manuscript. KB generated the constructs. RK, MT, MG, JP, RG, JK, BB, and FG performed experiments. MS revised the manuscript and provided mice. PC revised the manuscript and discussed data.

## Conflict of Interest Statement

All authors are employees of Bavarian Nordic GmbH, which funded the study, except for MS who is a consultant for Bavarian Nordic GmbH. The MVA used for this study was MVA-BN^®^. MVA-BN^®^ is Bavarian Nordic’s proprietary and patented technology.
