# Supplementary material for: NLRC4 Inflammasome-Driven Immunogenicity of a Recombinant MVA Mucosal Vaccine Encoding Flagellin
Source: Front Immunol. 2018 Jan 24;8:1988. doi: 10.3389/fimmu.2017.01988 (PMC5787573; doi:10.3389/fimmu.2017.01988)

Supplementary Figure 1. Efficacy of DT depletion. C57BL/6 and CD11c-DTR mice were treated *i.n.* with DT or PBS. The following day, lung cells were isolated and stained for DCs. (a) Frequency of cDCs identified as CD11c^hi^MHC-II^hi^ cells. (b) Frequency of CD103^+^CD11b^-^ and CD103^-^CD11b^+^ cDCs subsets in control and DT-treated wt and CD11c-DTR mice, gated on CD11c^hi^MHC-II^hi^ cDCs. (c) Frequency of CD172a^-^CD11b^-^ and CD172a^+^CD11b^+^ cDCs subsets in control and DT-treated wt and CD11c-DTR mice, gated on CD11c^hi^MHC-II^hi^ cDCs. cDCs cells were previously gated on Live/dead^-^CD19^-^CD3^-^ cells.


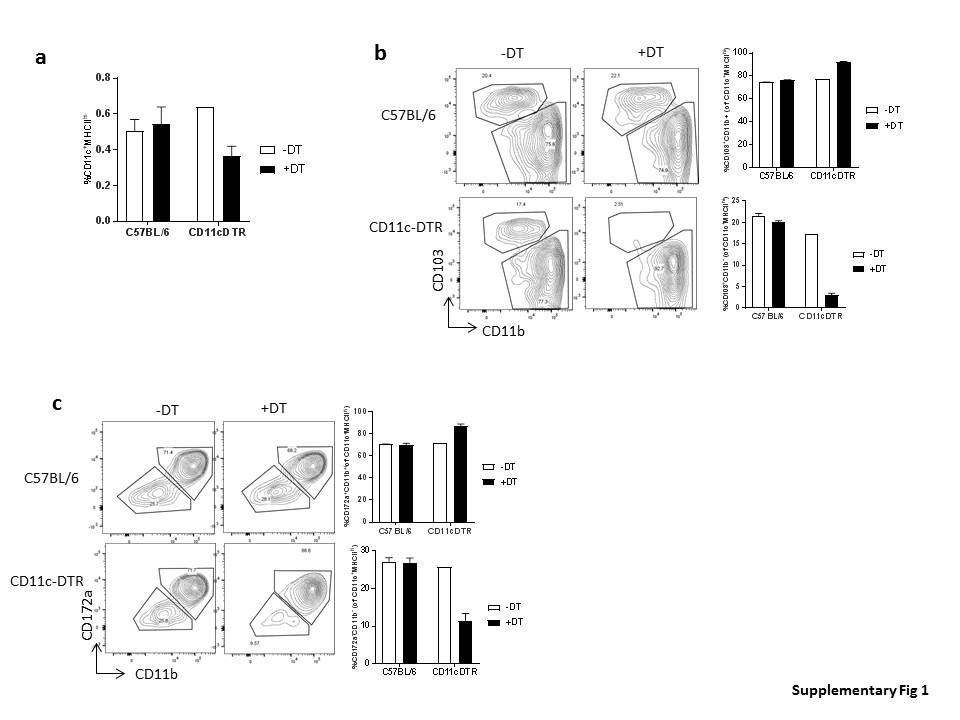

Supplement: Supplementary file 1 [file Data_Sheet_1.DOCX]
